# Identification and characterization of the anti-SARS-CoV-2 activity of cationic amphiphilic steroidal compounds

**DOI:** 10.1080/21505594.2022.2085793

**Published:** 2022-06-22

**Authors:** Alexandre Borin, Laís D. Coimbra, Karina Bispo-dos-Santos, Fabrício F. Naciuk, Marina Fontoura, Camila L. Simeoni, Giovanni V. Gomes, Mariene R. Amorim, Humberto D. Gravina, Jacqueline Farinha Shimizu, Amanda S. C. Passos, Isadora M. de Oliveira, Ana Carolina de Carvalho, Alisson Campos Cardoso, Pierina L. Parise, Daniel A. Toledo-Teixeira, Giuliana E. Sotorilli, Gabriela F. Persinoti, Ingra Morales Claro, Ester C. Sabino, Marcos R. Alborghetti, Silvana A. Rocco, Kleber G. Franchini, William M. de Souza, Paulo S. L. Oliveira, Thiago M. Cunha, Fabiana Granja, José Luiz Proença-Módena, Daniela B.B. Trivella, Marjorie Bruder, Artur T. Cordeiro, Rafael Elias Marques

**Affiliations:** a Brazilian Biosciences National Laboratory - LNBio, Brazilian Center for Research in Energy and Materials - CNPEM, Campinas, Brazil; b Department of Genetics, Evolution, Microbiology and Immunology, Institute of Biology, University of Campinas (UNICAMP), Campinas, Brazil; c Laboratory of Emerging Viruses (LEVE), Department of Genetics, Evolution, Microbiology and Immunology, Institute of Biology, University of Campinas (UNICAMP), Campinas, Brazil; d Department of Cellular and Structural Biology, Institute of Biology, University of Campinas (UNICAMP), Campinas, Brazil; e Center for Research in Inflammatory Diseases (CRID), Ribeirao Preto Medical School, University of Sao Paulo, Ribeirao Preto, Brazil; f Brazilian Biorenewables National Laboratory (LNBR), Brazilian Center for Research in Energy and Materials (CNPEM), São Paulo, Brazil; g Instituto de Medicina Tropical, Faculdade de Medicina da Universidade de São Paulo, Sao Paulo, Brazil; h World Reference Center for Emerging Viruses and Arboviruses and Department of Microbiology and Immunology, University of Texas Medical Branch, Galveston, TX, USA; i Biodiversity Research Center, Federal University of Roraima (UFRR), Boa Vista, Brazil; j Experimental Medicine Research Cluster, University of Campinas (UNICAMP), Campinas, Brazil

**Keywords:** SARS-CoV-2, drug discovery, steroidal compounds, antiviral activity

## Abstract

The ongoing COVID-19 pandemic caused a significant loss of human lives and a worldwide decline in quality of life. Treatment of COVID-19 patients is challenging, and specific treatments to reduce COVID-19 aggravation and mortality are still necessary. Here, we describe the discovery of a novel class of epiandrosterone steroidal compounds with cationic amphiphilic properties that present antiviral activity against SARS-CoV-2 in the low micromolar range. Compounds were identified in screening campaigns using a cytopathic effect-based assay in Vero CCL81 cells, followed by hit compound validation and characterization. Compounds LNB167 and LNB169 were selected due to their ability to reduce the levels of infectious viral progeny and viral RNA levels in Vero CCL81, HEK293, and HuH7.5 cell lines. Mechanistic studies in Vero CCL81 cells indicated that LNB167 and LNB169 inhibited the initial phase of viral replication through mechanisms involving modulation of membrane lipids and cholesterol in host cells. Selection of viral variants resistant to steroidal compound treatment revealed single mutations on transmembrane, lipid membrane-interacting Spike and Envelope proteins. Finally, in vivo testing using the hACE2 transgenic mouse model indicated that SARS-CoV-2 infection could not be ameliorated by LNB167 treatment. We conclude that anti-SARS-CoV-2 activities of steroidal compounds LNB167 and LNB169 are likely host-targeted, consistent with the properties of cationic amphiphilic compounds that modulate host cell lipid biology. Although effective in vitro, protective effects were cell-type specific and did not translate to protection in vivo, indicating that subversion of lipid membrane physiology is an important, yet complex mechanism involved in SARS-CoV-2 replication and pathogenesis.

## Introduction

The coronavirus disease 2019 (COVID-19) pandemic caused by severe acute respiratory syndrome coronavirus 2 (SARS-CoV-2) is the most important public health emergency in the last hundred years [1]. SARS-CoV-2 has caused drastic socioeconomic consequences worldwide and is responsible for more than 6.3 million deaths and 531 million cases as of June 2022 [2].

SARS-CoV-2 is an enveloped betacoronavirus with approximately 120 nm in diameter containing a single-stranded positive sense RNA genome. SARS-CoV-2 binds to the angiotensin-converting enzyme 2 (ACE2) receptor on the surface of host cells and is internalized and fuses with the endosome membrane to release infectious RNA into the cytoplasm [3]. After genomic and subgenomic RNAs are translated into non-structural and structural proteins, the viral genome is replicated and organized in newly formed viral particles. During this process, the viral M, S, and E proteins embedded in the host ER membrane provide the assembling structure to form the envelope around the cytosolic complex of genome and nucleocapsid proteins (N). Viral particles bud from the endoplasmic reticulum (ER), are transported through the Golgi complex and secreted from infected cells. The entire SARS-CoV-2 replication process is dependent on membranous structures, lipids, and transmembrane proteins, which are subverted during viral replication, creating “viral factories” [4]. Not surprisingly, changes in cellular lipid composition and distribution are also important for replication of other enveloped viral pathogens, and for triggering antiviral immune responses [5].

Monoclonal antibodies targeting cytokines and other effector molecules of the immune system were recently shown to be protective in patients with moderate-to-severe COVID-19 [6]. Unfortunately, effective antiviral treatments against SARS-CoV-2 are still lacking. Steroidal compounds are well known for their hormonal activity, as well as for their anti-inflammatory properties, the latter having proven effective in treating severe COVID-19 patients, as being the case for the corticosteroid dexamethasone [7]. Progesterone has also entered clinical trials as a possible therapy against COVID-19 [8] and has been shown to affect SARS-CoV-2 replication [9]. Herein, we describe two steroidal compounds, LNB167 and LNB169, that inhibit SARS-CoV-2 replication in different cell lines, presenting EC50 values in the low micromolar range. LNB167 and LNB169 are cationic amphiphilic compounds. Mechanistic studies indicate that LNB167 and LNB169 may affect cellular lipid membranes that are subverted by SARS-CoV-2 during viral replication, resulting in a reduced viral progeny.

## Materials and methods

### Cell lines

Vero CCL81 (BCRJ, # 0245), HEK-293T (provided by Sandra Dias – CNPEM, Brazil), HuH 7.5 (provided by Dr Claudia Santos, Fiocruz-PR, Brazil) were cultured in DMEM supplemented with 10% fetal bovine serum (FBS), 1% L-Glutamine, and 1% penicillin/streptomycin. Caco-2 (BCRJ, # 0059), Calu-3 (provided by Patrícia R. M. Rocco – Federal University of Rio de Janeiro, Brazil) were maintained in DMEM/Ham’s F-12 1:1 with 20% FBS 1% L-glutamine and 1% penicillin/streptomycin.

### Virus and preparation of viral stocks

The SARS-CoV-2 strain HIAE-02 SARS-CoV-2/SP02/human/2020/BRA (GenBank accession number MT126808.1) isolated in Brazil (provided by Prof. Edison Luiz Durigon, USP-SP, Brazil) was propagated in Vero CCL81 cells. Supernatants were collected after 36–40 h post-infection, stored in aliquots at −80°C and titrated using plaque assays. All assays with viable SARS-CoV-2 were performed in a BSL-3 facility located in the Laboratory of Emerging Viruses (LEVE) at the University of Campinas.

### Compounds

Compounds were synthesized as described in (10.1021/acsmedchemlett.0c00106).

### High-throughput screening (HTS)

#### SARS-CoV-2 cytopathic effect and cell viability assays

For evaluation of cytopathic effect induced by SARS-CoV-2 in Vero CCL81, cells were seeded in two 384-well microplates at a concentration of 1.7 × 10^3^ cells per well and incubated overnight at 37°C 5% CO2 for attachment. Next, compounds were transferred to both plates to a final concentration of 20 µM (0.4% DMSO). One of the plates was then infected with SARS-CoV-2 at a multiplicity of infection (MOI) of 0.1, while the other received medium. Both plates were then incubated for 60 h at 37°C 5% CO2. After the period of incubation, cells were stained during 45 min with 2 µM Hoechst -33,342 and 100 nM Mitotracker Deep Red and fixed in 4% w/v PFA in PBS. For concentration response curves, compounds were serially diluted in nine points with a 1/2 dilution factor. Final assay concentrations ranged from 40 to 0.156 µM.

#### Imaging and data processing

Cell images were acquired using an Operetta automated microscopy (Perkin Elmer). To evaluate SARS-CoV-2-induced cytopathic effect, we quantified the number of nuclei and mitochondrial signal intensity with 10X and 20x objective lenses. The percentual of inhibition of SARS-CoV-2-induced cytopathic effect in a given experimental group was calculated by correlation, using vehicle-treated non-infected wells as positive controls and vehicle-treated SARS-CoV-2-infected wells as negative controls.

### Virus and preparation of viral stocks

The SARS-CoV-2 strain HIAE-02 SARS-CoV-2/SP02/human/2020/BRA (GenBank accession number MT126808.1) isolated in Brazil (provided by Prof. Edison Luiz Durigon, USP-SP, Brazil) was propagated in Vero CCL81 cells. Supernatants were collected after 36–40 h post-infection, stored in aliquots at −80°C and titrated using plaque assays. All assays with viable SARS-CoV-2 were performed in a BSL-3 facility located in the Laboratory of Emerging Viruses (LEVE) at the University of Campinas.

### Compounds

Compounds were synthesized as described in (10.1021/acsmedchemlett.0c00106).

### High-throughput screening (HTS)

#### SARS-CoV-2 cytopathic effect and cell viability assays

For evaluation of cytopathic effect induced by SARS-CoV-2 in Vero CCL81, cells were seeded in two 384-well microplates at a concentration of 1.7 × 10^3^ cells per well and incubated overnight at 37°C 5% CO2 for attachment. Next, compounds were transferred to both plates to a final concentration of 20 µM (0.4% DMSO). One of the plates was then infected with SARS-CoV-2 at a multiplicity of infection (MOI) of 0.1, while the other received medium. Both plates were then incubated for 60 h at 37°C 5% CO2. After the period of incubation, cells were stained during 45 min with 2 µM Hoechst -33,342 and 100 nM Mitotracker Deep Red and fixed in 4% w/v PFA in PBS. For concentration response curves, compounds were serially diluted in nine points with a 1/2 dilution factor. Final assay concentrations ranged from 40 to 0.156 µM.

#### Imaging and data processing

Cell images were acquired using an Operetta automated microscopy (Perkin Elmer). To evaluate SARS-CoV-2-induced cytopathic effect, we quantified the number of nuclei and mitochondrial signal intensity with 10X and 20x objective lenses. The percentual of inhibition of SARS-CoV-2-induced cytopathic effect in a given experimental group was calculated by correlation, using vehicle-treated non-infected wells as positive controls and vehicle-treated SARS-CoV-2-infected wells as negative controls.

### Compound validation assays

#### Antiviral activity assay

Cells were seeded in 24-well plate at 2.0 × 10^5^ cells per well for Caco-2 and Calu-3, 2.5 × 10^5^ for Vero CCL81 and HuH7.5 and 3.5 × 10^5^ for HEK293T for antiviral compound validation. After 24 h, cells were infected with SARS-CoV-2 at an MOI of 0.1 for HEK293T, HuH7.5, Caco-2, and Calu-3 or 0.01 for Vero CCL81 during 1 h. After infection, cells were treated with vehicle (DMSO) or compounds at concentrations of 10 µM or 32 µM for 48 h. Supernatant samples were collected to assess SARS-CoV-2 titers using plaque assay (infectious viral particle) and RT-qPCR (viral RNA).

#### MTT assay

Compound toxicity was evaluated using the MTT assay in a 24-wells plate format for cell lines Vero CCL81, HEK293T, HuH7.5, Caco-2, and Calu-3. Compounds were added at various concentrations and removed after 48 h of incubation. The MTT reagent (3-(4,5-dimethylthiazol-2-yl)-2,5-diphenyl tetrazolium bromide) was added to cell cultures and incubated for 3 h at 37°C in 5% CO2. After substrate removal, cells were solubilized in DMSO, and absorbance was measured at 490 nm using a spectrophotometer with a plate reader. The percentage of cell viability for each group was normalized to absorbance levels from non-treated control cells.

#### Viral RNA extraction and quantification by RT-qPCR

Viral RNA from cell culture supernatants were extracted using PureLink RNA Mini Kit (Invitrogen), following the manufacturer’s protocol, and verified by spectrophotometry in Nanodrop One (ThermoFisher Scientific). SARS-CoV-2 RNA detection and quantification was performed with specific primers and probe for the gene coding for its envelope protein (Forward primer: 5’- ACAGGTACGTTAATAGTTAATAGCGT-3“+, Reverse primer: 5’-ATATTGCAGCAGTACGCACACA-3”, Probe: 5’−6FAM-ACACTAGCCATCCTTACTGCGCTTCG-QSY-3“+), according to Charité’s One-step RT-qPCR protocol [10]. Extracted RNA was 100-fold diluted in ultrapure water, and 6 µL of dilution was used for each reaction, along with 3 µL of TaqMan Fast Virus 1-Step Master Mix (applied Biosystems), 800 nM of each primer, and 400 nM of probe. Data acquisition was conducted in QuantStudio3 System (Applied Biosystems) in samples and control samples through the following cycling algorithm: 1 cycle of 50°C for 10 min, 1 cycle of 95°C for 2 min, followed by 45 cycles of 95°C for 5 s and 60°C for 30 s.

#### Virus plaque assay

SARS-CoV-2 infectious virus particles were measured by plaque assay in Vero CCL81 cells in 24-well plates. Supernatants collected from assays were serially diluted, added to Vero CCL81 confluent monolayers and incubated for 1 h at 37°C 5% CO2. Samples were removed, and cell cultures were covered with a semi-solid overlay medium (1% w/v carboxymethyl cellulose in DMEM supplemented with 5% FBS) and incubated for 4 days. Cells were fixed in 8% w/v paraformaldehyde for 1 h and stained with a 1% w/v methylene blue solution. Viral titers were calculated by counting the lysis plaques and corrected by the sample dilution factor and volume. SARS-CoV-2 viral titer results were expressed as plaque-forming units per mL of supernatant (PFU/ml).

#### Cholesterol staining assay

A 96-wells plate was seeded with 1 × 104 Vero CCL81 cells per well. After 24 h, cells were treated with U18666A (at 10 µM) or LNB167 (at 32 µM or 10 µM), infected or not with SARS-CoV-2 (MOI 0.01) and incubated for 24 h at 37°C 5% CO2. Cell nuclei were stained in green with SYBR Safe DNA Gel Stain dye (Thermo Fisher), and the cholesterol was stained in blue with the reagent Filipin III from Streptomyces filipinensis (Sigma Aldrich). Cell imaging was performed at the Operetta automated microscopy with 20X objective lens, as described above.

### Time of addition

The time of addition assay was performed in 24-well plates plated with 2.5 × 10^5^ Vero cells per well. Cell cultures were infected with SARS-CoV-2 B.1 (MOI 0.01) at a moment defined as time 0 h (t0). Next, 12 subsequent time points were established in 2 h intervals in which compounds were added, up to 24 h post-infection. Every time point (2 h, 4 h, 6 h, 8 h, 10 h, 12 h, 14 h, 16 h, 18 h, 20 h, 22 h, 24 h) included 4 technical replicates of cell cultures treated with LNB167 or LNB169 at a concentration of 32 µM. Supernatant samples from each time point were collected 24 h post-infection for viral load assessment using plaque assays and RT-qPCR.

### Virucidal assay

The virucidal assay was performed by incubating 103 SARS-CoV-2 PFU with LNB167 or LNB169 at a concentration of 32 µM diluted in DMEM without SFB for 1 h at 37ºC. A subsequent plaque assay was performed to assess the sample viral load.

### SARS-CoV-2 genome analysis

#### Resistant variant and cross-protection assay

To induce a resistant viral variant, SARS-CoV-2 B (PANGO lineage) was cultured in Vero CCL81 in the presence of compounds for 48 h. We performed eight consecutive passages with increasing concentrations of compounds. After 48 h, supernatant was collected for assessment of infective viral particles using plaque assay. The first two passages occurred with LNB167 and LNB169 at a concentration of 3 µM. The following three passages were performed with compounds at 10 µM and the last set of three passages occurred with compounds at 32 µM. After eight passages, the samples of SARS-CoV-2 B produced in presence of LNB167 and LNB169 were named SARS-CoV-2 167 R or SARS-CoV-2 169 R, respectively. Samples were titrated, sequenced, and used in cross-protection experiments. Cross-protection assays consisted in infecting Vero CCL81 cell cultures with SARS-CoV-2 169 R at a MOI 0.01 and treating with LNB167 at 32 µM for 48 h. Supernatant samples were collected and assessed using RT-qPCR and plaque assay.

#### Viral RNA genome sequencing

SARS-CoV-2 genome sequencing was carried out using the MinION sequencing platform (Oxford Nanopore Technologies, https://nanoporetech.com) and the ARTIC version 3 protocol (https://nanoporetech.com) and metagenomic using SMART-9N amplification using with MinION sequencing (Oxford Nanopore Technologies, Oxford, UK), as described elsewhere [11]. A multiplex PCR approach was used as previously described [12,13] for whole-genome amplification, using ARTIC V3 primer schemes. The PCR product was purified using AMPure XP magnetic beads (Beckman Coulter, Inc., https://nanoporetech.com), according to manufacturer’s instructions. Qubit dsDNA High Sensitivity assay was used for DNA quantification with the Qubit 3.0 (Thermo Fischer Scientific, USA). Library preparation started with equimolar normalization of 10 ng per sample, which were barcoded using the EXP-NBD104 Native Barcoding Kits (Oxford Nanopore Technologies), and SQK-LSK109 Kit (Oxford Nanopore Technologies). The library was loaded onto an R9.4.1 flow-cell (Oxford Nanopore Technologies) and sequenced using MinKNOW version 20.10.3 (Oxford Nanopore Technologies). The resulting FAST5 files were basecalled, demultiplexed, and trimmed using Guppy version 4.4.1 (Oxford Nanopore Technologies). The reads were aligned against the reference genome Wuhan-Hu-1 (GenBank accession no. MN908947) using Minimap2 version 2.17.r941 [14] and converted to a sorted BAM file using SAMtools [15]. Length filtering, quality test, and primer trimming were performed for each barcode using Guppyplex from ARTIC (https://nanoporetech.com). Genome regions with a depth of <20-fold were represented with N ambiguity character. Sequence coverage for the SARS-CoV-2 parent strain was 99.6%, 91.9% for 167 R, and 79.7% for 169 R. 167 R and 169 R sequences were aligned with their original reference sequences and analyzed with Mafft v.7.375 [16] and visualized in Geneious Prime software (Biomatters, Ltd, New Zealand).

#### Illumina mRNA library preparation

For Illumina library preparations, 1.5 µg of total RNA of each sample were processed using the TruSeq Stranded mRNA LT Sample Prep Kit from Illumina (Illumina Inc.) according to the manufacturer’s protocol. The size and quality of libraries were validated on an Agilent Bioanalyzer 2100 with the 12,000 DNA assay kit (Agilent) and quantified through qPCR using the KAPA Library Quantification Kit Illumina® Platforms (Kapa Biosystems). The libraries were pooled in equimolar ratios and subsequently submitted to paired-end sequencing on the NovaSeq instrument (Illumina Inc.), available at the Genohub Next-Generation Sequencing Services (USA).

#### Bioinformatic analysis

Raw RNAseq reads were quality checked using FastQC (https://nanoporetech.com) and filtered using BBDuk to remove low quality reads and adapter sequences. QC reads were subject to SortmeRNA v.4.2.0 [17] to remove rRNA reads. Filtered reads were aligned to the human (hg38) and SARS-CoV-2 (GCA_011537625) genomes available at NCBI using Histat2 [18] in strand-specific mode. The featureCounts function from Rsubread package [19] was used to count mapped reads and assign them to transcripts. Reproducibility among biological replicates was assessed by exploring a PCA of the top 500 genes that have the largest variation, as implemented in the plotPCA function from DESeq2 package, which led to the removal of one replicated from SARS_LNB condition. Differential expression analysis was performed using DESeq2 [20]. Genes with adjusted *p*-values <0.05 were considered differentially expressed (DEG). Gene Ontology (GO) enrichment analysis and dot plots were performed using the cluster Profiler R package [21].

### In vivo experiments

#### Pharmacokinetics in mice

*In vivo* pharmacokinetics assessment of LNB167 was performed at the Center of Innovation and Preclinical Studies (CIEnP, Brazil) under contract. LNB167 was tested in adult male CD1 mice (n = 5) bred under Specific Pathogen Free conditions at doses of 60 mg/kg (p.o.) or 6 mg/kg (I.V.). Results were reported according to ARRIVE (Animal Research Reporting In Vivo Experiments) [22–24]. The pharmacokinetics parameters were determined using the software Phoenix® WinNonlin® (version 7.0).

#### Potential toxicity of LNB167 in mice

Adult FVB male mice bred and maintained at the Animal Facility of LOM at LNBio/CNPEM were weighed and divided into three groups (n = 5): vehicle (5% ethanol, 45% PEG400, 5% TWEEN®80, 45% saline), LNB167 60 mg/kg, and LNB167 300 mg/kg. Mice received one dose daily for 6 days by oral gavage. Animals were weighed and monitored daily for signs of potential compound toxicity including loss of movement, ruffled fur, ear position, eye shape, and death.

#### In vivo experimental treatment

Female K18-hACE2 mice [25–27], 8 to 10-weeks-old, were inoculated with 2 × 104 PFU of SARS-CoV-2 (40 µL) intranasally. Uninfected mice received sterile saline solution intranasally. Infected mice were treated once daily with LNB167 (120 mg/kg) (N = 6) or vehicle (N = 6) from day 0 to day 3 p.i. Uninfected mice also received LNB167 (N = 5) or vehicle (N = 5). Body weight was evaluated on the baseline and on all the days post-infection. On the day 3 p.i., animals were humanely euthanized, and lungs were collected. The right lung was harvested and homogenized in 1 mL of ice PBS and stored at −70ºC for posterior viral titration by RT-qPCR or by Virus plaque assay. The left lung was collected in paraformaldehyde (PFA 4%), Posteriorly, paraffin-embedded slices (5 µm) were stained with hematoxylin and eosin (H&E) for histological evaluation. Photomicrographs were taken in 10x and 20x magnitude lens using a Leica DMI 6000 B microscopy.

All the in vivo experimental procedures were performed in a biosecurity laboratory level 3 and in accordance with the guide for the use of laboratory animals of the University of Sao Paulo and approved by the institutional ethics committee under the protocol number 1011/2021.

### Statistical analyses

Datasets from high-content imaging experiments were validated using the Z-factor calculated from technical replicates in non-infected and infected controls. Z-factors above 0.5 were considered adequate. For dose–response curves, data were normalized and fitted using non-linear regression. Results from plaque assays and RT-qPCR tests were analyzed using non-parametric Kruskal–Wallis test coupled to Dunn’s multiple comparison test. All analyses were performed using GraphPad Prism v8.4.3.

## Results

### Identification of cationic amphiphilic steroidal compounds with anti-SARS-CoV-2 properties in vitro

**Figure 1.**
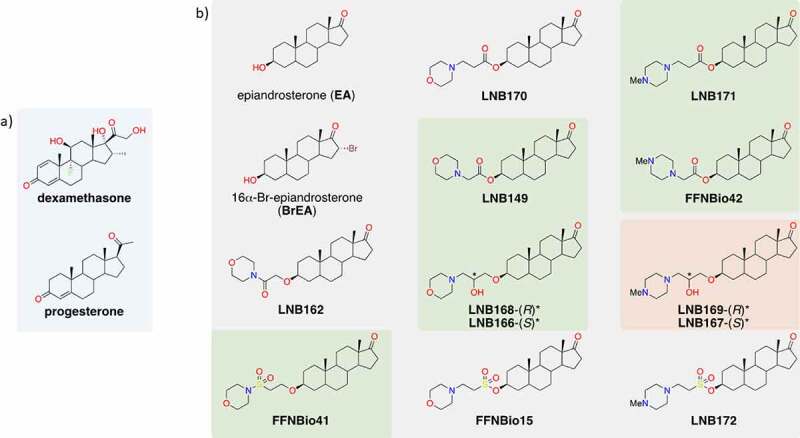
Chemical structure of selected steroidal compounds. (a) Chemical structures of dexamethasone and progesterone, which are currently used or evaluated, respectively, in clinical settings for treating COVID-19; (b) Chemical structures of epiandrosterone (EA) derivatives evaluated in this study. Compounds showing high to moderate SARS-CoV-2 CPE inhibition are highlighted in green rectangles and the best compounds, LNB167 and LNB169 are highlighted in a red rectangle.

**Figure 2.**
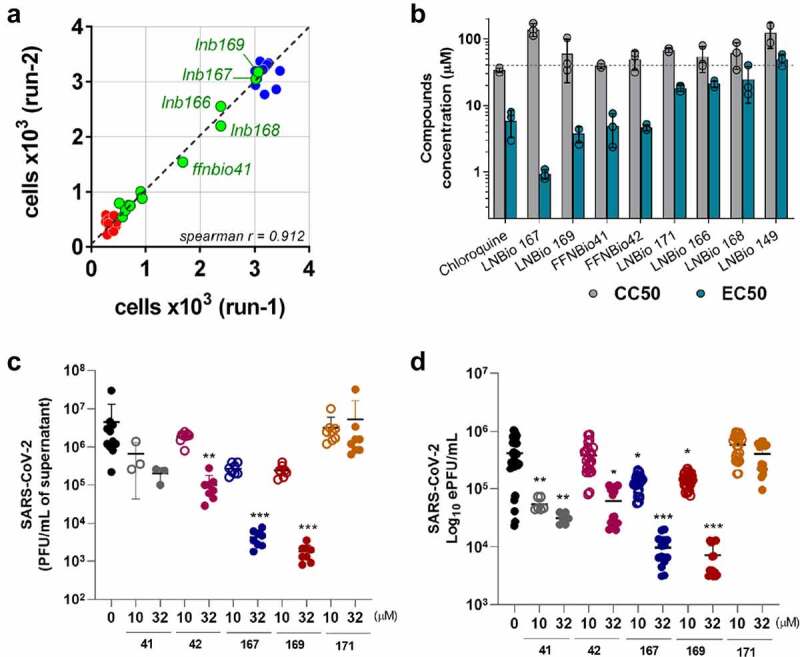
Identification of steroidal compounds with anti-SARS-CoV-2 properties in vitro. (a) Correlation plot between two independent runs of the SARS-CoV-2 mediated cytopathic effect (CPE) in Vero cells assay. Control groups were defined by non-infected (blue circles) and infected (red circles) cells. Among the twelve assayed compounds (green circles), five promoted a significant cellular protective effect and were selected as hit candidates (labeled samples). (b) Half maximal effective concentration (EC50) (green bar) was calculated based on 50% CPE inhibition and half maximal cytotoxic concentration (CC50) (gray bar) corresponds to the compound concentration that causes 50% of cytotoxicity in non-infected cells. Dots represent mean of triplicates in one experiment. Bars represent 3 independent experiments. (c) SARS-CoV-2 infectious viral particles quantification in Vero cells supernatant infected with or without treatment by plaque assay titration. (d) SARS-CoV-2 RNA quantification by RT-qPCR in supernatant of infected cells with or without treatment. *p < 0.05, **p < 0.01, ***p < 0.001 relative to the virus-infected, vehicle-treated control group. Vehicle = DMSO. Data representative of 2 independents experiments. n = 3 per experiment.

We performed a phenotypic screening assay in Vero CCL81 cells infected with SARS-CoV-2 in a biosafety level 3 (BSL3) facility, leading to the identification of a series of compounds with cytoprotective properties against SARS-CoV-2 infection (Figure 1). Screening campaigns were performed at a fixed 20 µM compound concentration in which 5 out of 12 heterocycle-containing steroidal compounds derived from epiandrosterone (EA) were classified as hits, inhibiting SARS-CoV-2-induced cytopathic effect (CPE) in cell culture by at least 50% (Figure 2(a)). Concentration–response curves performed with our 5 hit compounds plus 3 related compounds indicated that LNB167, LNB169, FFNBio41, and FFNBio42 had a half maximal effective concentration (EC50) value in the low micromolar range (0.9 µM, 3.8 µM, 5.0 µM, and 4.7 µM, respectively), whereas compounds LNB149, LNB166, LNB168, and LNB171 had EC50 values in the mid micromolar range. Noteworthy, the three best compounds all share an *N*-methylpiperazine ring linked to the androstane core by either 2 or 3 carbons, whereas less potent compounds displayed a morpholine ring. As such, these hits could be classified as cationic amphiphilic compounds, since they presented a hydrophobic steroid core, and a polar protonated moiety at physiological pH. At the time (early 2020), several articles had already shown the antiviral properties of such molecules [28]. Chloroquine, which is known to present anti-SARS-CoV-2 activity in vitro in Vero CCL81 cells [29] also showed an EC50 value of 5.9 µM in our assays. Hit compounds were nontoxic in Vero CCL81 cells in most concentrations tested, with half maximal cytotoxic concentrations (CC50) values above 40 µM (Figure 2(b), Table S1).

Compound LNB167 was the most potent and also the less toxic in the series, resulting in a selectivity index (SI) of 154 (Table S1).

We selected compounds FFNBio41, FFNBio42, LNB167, LNB169, and LNB171 to test for potential antiviral activities against SARS-CoV-2 in Vero CCL81 cell cultures. Cell monolayers were infected with SARS-CoV-2 and then incubated with compounds at concentrations of 10 or 32 µM until sample collection at 48 h post-infection (Figure 2(c,d)). Viral load was assessed in supernatant samples by viral plaque assay (Figure 2(c)), which indicates infectious viral progeny, and by RT-qPCR (Figure 2(d)), which indicates levels of viral RNA. Treatment with compound FFNBio41 had no effect on the viral load assessed by plaque assays (Figure 2(c)) but led to a 10-fold reduction in levels of viral RNA in both concentrations tested, assessed by RT-qPCR (Figure 2(d)). Treatment with FFNBio42 at 32 µM caused a 10-fold reduction in viral load in comparison to the vehicle-treated control using both methods for viral load assessment. Treatment with compounds LNB167 or LNB169 resulted in potent inhibition of SARS-CoV-2 replication, reducing levels of SARS-CoV-2 in both concentrations tested and methods used. Treatment with LNB167 or LNB169 at 32 µM led to a 1000-fold reduction in infectious virus and almost 100-fold reduction in viral RNA levels in cell culture supernatant, in comparison to vehicle-treated controls (Figure 2(c,d)). Despite causing a reduction of SARS-CoV-2-induced CPE in screening assays, treatment with LNB171 had no effect on viral load in any of the tested conditions. Cell viability experiments using the MTT assay indicated that steroidal compounds were not toxic to Vero CCL81 cells at the tested concentrations (Figure S1A).

In summary, we selected compounds LNB167 and LNB169 for further characterization due to their potency in inhibiting SARS-CoV-2 replication and favorable toxicity profile in Vero CCL81 cells.

### Compounds LNB167 and LNB169 reduce SARS-CoV-2 replication in human cell lines

**Figure 3.**
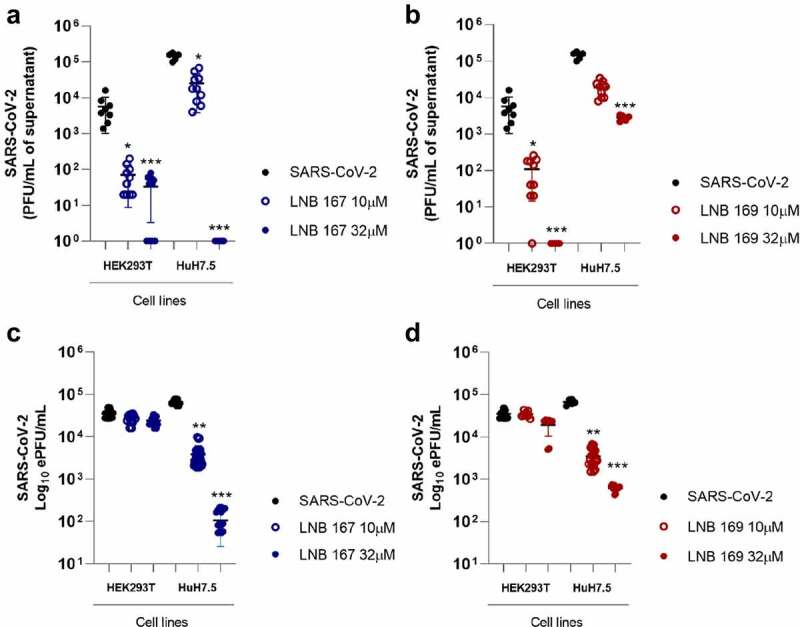
LNB167 and LNB169 have antiviral effects against SARS-CoV-2 in different cell lines. (a, b) Quantification of SARS-CoV-2 infectious viral particles in the supernatant of different cell lines (HEK293T and HuH7.5) infected with SARS-CoV-2 with or without treatment, using plaque assays. (c, d) Viral RNA measured by RT-qPCR in supernatant of cell cultures of different cell lines infected with or without LNBio167 and LNB169 treatment. *p < 0.05, **p < 0.01, ***p < 0.001 relative to the virus-infected, vehicle-treated control group. Vehicle = DMSO. Data representative of 2 independent experiments (n = 6).

We moved on to test the antiviral activity of compounds LNB167 and LNB169 against SARS-CoV-2 in four different human cell lines: HEK293T, HuH7.5, Caco-2, and Calu-3 (Figure 3 and Figure S2). Supernatant samples were collected from SARS-CoV-2-infected cultures treated or not with LNB167 or LNB169 and viral load was assessed by plaque assay (Figure 3(a,b)) and by RT-qPCR (Figure 3(c,d)). Our results indicated that treatment with both compounds resulted in significant decreases in viral load in HEK293T and HuH7.5 cells, which were more pronounced when assessing infective viral progeny by plaque assays. Treatment with LNB167 at 10 µM reduced SARS-CoV-2 viral load in 10 to 100-fold, whereas treatment at 32 µM resulted in a 100-fold reduction in viral load in HEK293T cells and a striking 100.000-fold reduction in viral load recovered from HuH7.5 cells (Figure 3(a)). Compound LNB169 showed similar results, reducing viral load in HEK293T and HuH7.5 in approximately 10 to 100-fold at 10 µM and up to a 10.000-fold in HEK293T cells at 32 µM concentration (Figure 3(b)). Quantification by RT-qPCR indicated that treatment with LNB167 and LNB169 did not reduce levels of viral RNA in HEK293T cells (Figure 3(c,d)), while in HuH7.5 cells, both compounds caused reductions in viral RNA levels. Treatment with LNB167 caused up to a 1000-fold reduction in viral load at 32 µM (Figure 3(c)) and treatment with LNB169 reduced viral load by approximately 100-fold also at 32 µM (Figure 3(d)). Compounds LNB167 and LNB169 at both concentrations had no effect on the SARS-CoV-2 viral load in Caco-2 and Calu-3 human cell lines (Figure S2). Cell viability assessed using the MTT assay indicated that LNB167 and LNB169 were not toxic to either of the four cell lines tested (Figure S1B, C). Thus, our results show that treatment with LNB167 and LNB169 inhibit SARS-CoV-2 replication in human cell lines, but this protective effect is restricted to certain cell types.

### LNB167 and LNB169 affect the initial phases of SARS-CoV-2 replication

**Figure 4.**
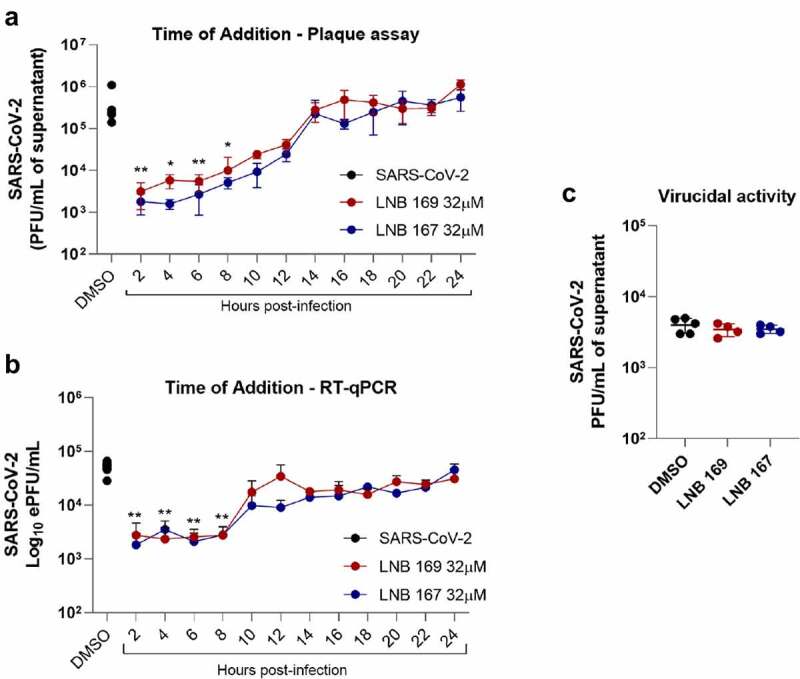
LNB167 and LNB169 act in early stage of replication cycle of SARS-CoV-2. (a) Titer of SARS-CoV-2 infectious viral particles measured after 24 h in Vero infection and treatment with LNB167 and LNB169 (32 µm). (b) SARS-CoV-2 RNA quantification by RT-qPCR in supernatant of Vero cells infected. (c) Compounds LNB167 and LNB169 were incubated with SARS-CoV-2 stock samples for 2 h at the concentration of 32 µm and later submitted to a plaque assay for quantification of infectious viruses in Vero CCL81 cells. Virus samples incubated with vehicle (DMSO) in cell culture media was included as a control. Results are expressed as PFU/mL of cell culture supernatant. *p* < 0.05, **p < 0.01, ***p < 0.001 in comparison an infected non-treated group. n = 4 for each time point.

We performed experiments to investigate possible antiviral mechanisms of action of compounds LNB167 and LNB169 against SARS-CoV-2 in vitro. First, we performed a time-of-addition study using Vero CCL81 cells, in which compounds were added after virus adsorption in 2 h intervals up to 24 h, which encompasses a full SARS-CoV-2 replication cycle. Supernatant samples were collected 24 h p.i. for viral load assessment using plaque assay and RT-qPCR (Figure 4). Both compounds inhibited SARS-CoV-2 replication in comparison to vehicle-treated controls, and both inhibited initial phases of the viral replication cycle, presenting maximum antiviral activity when added until 8 h after infection (Figure 4). Treatment with LNB167 or LNB169 resulted in up to 100-fold reduction in infectious viral progeny as assessed by plaque assays (Figure 4(a)), and in approximately 10-fold reduction in viral load assessed by RT-qPCR (Figure 4(b)). We also tested LNB167 and LNB169 for a potential virucidal activity, by incubating SARS-CoV-2 stock samples with compounds in DMEM for 2 h and subsequently assessing the infective viral load using plaque assays (Figure 4(c)). Our results indicated that neither LNB167 nor LNB169 were virucidal against SARS-CoV-2, as the infectious viral load was similar between treated and control groups. Moreover, this result also indicated that compounds do not affect SARS-CoV-2 particle integrity.

### Steroidal compounds elicited the emergence of SARS-CoV-2 variants with partial or complete resistance associated with mutations in structural proteins

**Figure 5.**
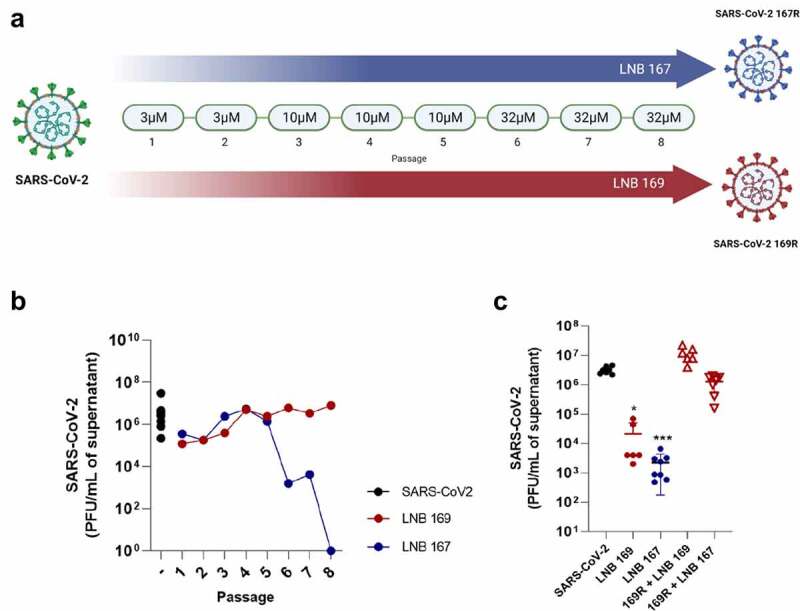
In vitro induction of LNB167 and LNB169-resistant SARS-CoV-2 variants. (a) Illustration of the serial passage program used to select LNB167 or LNB169-resistant SARS-CoV-2 variants in Vero CCL81 cells. (b) Titration of infectious viral particles of SARS-CoV-2 cultured in presence of LNB167 (blue circles) and LNB169 (red circles). Control infected non-treated group (black circle). Vehicle: DMSO. (c) Cross protection assay between SARS-CoV-2 169 R and treatment with LNB167 (32 µm). Infectious particles measured in supernatant of Vero cells infected with MOI of 0.01 after 48 h. Control SARS-CoV-2 B.1 infected non-treated (black circles); Control SARS-CoV-2 B.1 infected treated with LNB169 32 µm (red circle); Control SARS-CoV-2 infected treated with LNB167 32 µm (blue circle); Experimental group of resistant variant 169 R treated with LNB169 32 µm (red hollow upward triangle); Experimental group of resistant variant with 169 R infection and cross protect treatment with LNB167 32 µm (red hollow downward triangle). *p* < 0.05, **p < 0.01, ***p < 0.001 groups compared with control infected non-treated group (black circle).

We performed a series of in vitro SARS-CoV-2 passages in Vero CCL81 cells incubated with increasing concentrations of LNB167 or LNB169, to induce the emergence of compound-resistant viral variants. Control SARS-CoV-2 stocks were passaged in Vero CCL81 cells in media added with DMSO vehicle. The infectious viral load present in cell culture supernatants of each serial passage was assessed using plaque assays to indicate gain of fitness (Figure 5(a)). SARS-CoV-2 serial passages at 3 µM or 10 µM of either compound caused a detectable but discrete reduction in viral load (Figure 5(b)). Passage progression to the concentration of 32 µM in passage n°6 led to a dramatic decrease in viral load in the LNB167-treated passage but had no effect on the LNB169-treated passage. Repeated passages at 32 µM confirmed that viruses passaged in the presence of LNB167 had not acquired resistance, as the viral load continued to decrease (Figure 5(b)). Conversely, viruses passaged in LNB169 acquired resistance to the compound, and remained at high viral titers in subsequent passages at 32 µM. In order to investigate other aspects of viral resistance, we produced a stock of the SARS-CoV-2 LNB169-resistant variant (169 R) after passage n°8 and of the partially resistant SARS-CoV-2 variant 167 R after passage 5 (Figure 5(a,b)).

A cross-protection assay was performed to evaluate if the resistant variant 169 R was also resistant to compound LNB167. We infected Vero CCL81 cells with the conventional SARS-CoV-2 or with 169 R and added LNB167 or LNB169 at 32 µM concentration. Supernatant samples were collected 48 h p.i. for assessment of viral titers using plaque assays Conventional SARS-CoV-2 was susceptible to treatment with both steroidal compounds and showed 100 to 1000-fold reductions in viral load in comparison to vehicle-treated controls. 169 R-infected cultures treated with LNB169 showed no reduction in viral load, presenting viral loads similar to those in the vehicle-treated infected group. Moreover, 169 R-infected cultures showed no reduction in viral load when treated with LNB167, indicating that 169 R was cross-resistant to treatment with LNB167 (Figure 5(c)).

**Figure 6.**
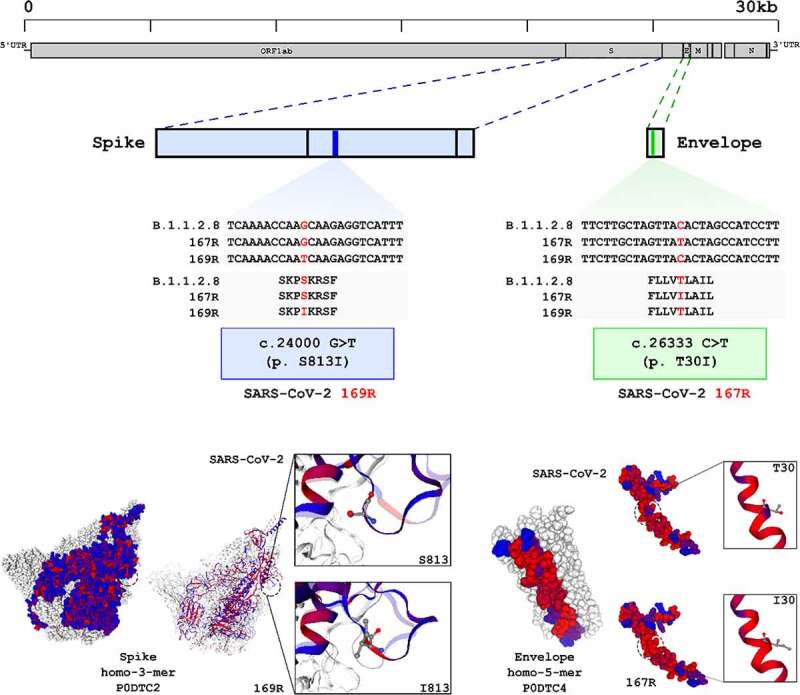
SARS-CoV-2 169R and 167R present single nucleotide mutations leading to amino acid substitutions in structural proteins. Genome sequencing from both SARS-CoV-2 and drug resistant variants showed that 169R and 167R present single nucleotide mutations. Mutation S813I is shown in 169R Spike protein S2 subunit, only 3 residues ahead of the internal fusion peptide. Variant 167R has a T30I mutation in its Envelope protein. Schemes indicate the mutation sites presented by each variant along their genomes and proteins. Structures from SARS-CoV-2 spike (entry: P0DTC2) and envelope proteins (entry: P0DTC4) were obtained at Protein Data Bank (PDB). Red dots represent hydrophobic amino acids, and blue dots represents hydrophilic amino acids.

Genome sequencing of variant 169 R, the partially resistant 167 R variant and the conventional SARS-CoV-2 parent strain was performed in search of mutations associated with resistance phenotypes (Figure 6). Sequences were obtained using a combination of the MinION platform and metagenomic techniques for a mean sequencing depth of 20-fold or greater. Using the sequence of conventional SARS-CoV-2 parent strain as reference, which was also passaged in Vero CCL81 cells, we observed one single nucleotide substitution in 169 R and another in 167 R, both in structural proteins (Figure 6). 169 R presented the single nucleotide substitution G>T in position 24,000, which translated to a change in serine 831 to an isoleucine near the internal fusion peptide in the Spike protein. 167 R presented a C>T mutation in position 26,333, resulting in an isoleucine in position 30 of the transmembrane domain of Envelope protein, instead of threonine found in the parental strain (Figure 6). Both substitutions resulted in changes from polar, uncharged amino acid residues to a hydrophobic isoleucine residue in structural proteins. Overall, we induced the emergence of complete or partially resistant viral variants against LNB169 and LNB167, respectively. Variants accumulated non-synonymous single mutations in structural proteins that are associated with lipid membranes in multiple steps during viral replication. As LNB169 induced a complete resistant variant over short passages, we continued our investigations only with LNB167, which induced mutations on structural proteins but without loss of compound efficacy.

### LNB167 affect SARS-CoV-2 replication by altering host cell lipid metabolism and cholesterol distribution

**Figure 7.**
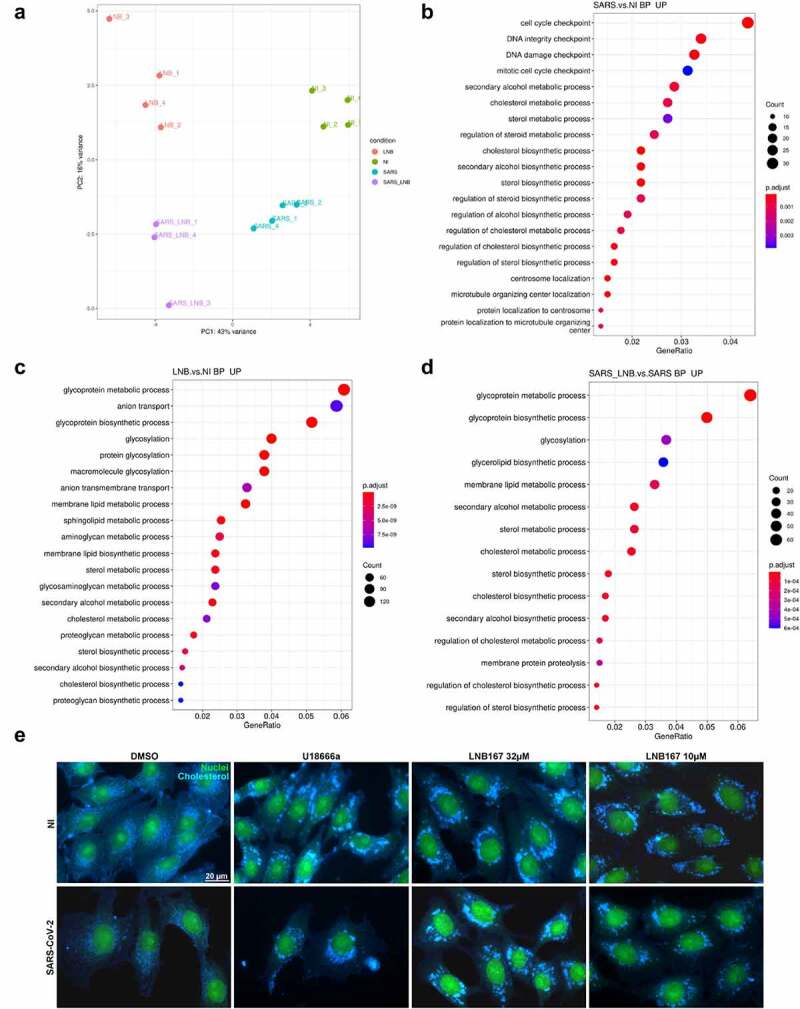
Treatment with LNB167 modulates lipid membrane and cholesterol metabolism. HEK293T cells were infected with SARS-CoV-2 and treated or not with LNB167 at 32 µm and collected at 48 h p.I. For RNASeq analysis. Non-infected (NI) HEK293T cells treated or not with LNB167 were included as controls. Gene expression data were analyzed using DESeq2 and Metacore in search of significant genes and pathways that were significantly up or downregulated in the tested conditions. (a) Principal Component Analysis (PCA) depicting global transcriptional profiles of tested conditions. (b) Enriched biological processes GO terms within upregulated genes in SARS-CoV-2 infected cells in comparison to NI cells (c) Enriched biological processes GO terms within upregulated genes in LNB167- treated cells in comparison to vehicle-treated cells (NI). (d) Enriched biological processes GO terms within upregulated genes in SARS-CoV-2 infected cells treated with LNB167 compared to vehicle-treated infected cells (NI). E) Immunostaining experiment in which Vero CCL81 cells were infected or not with SARS-CoV-2 and treated with U18666A, a cholesterol intracellular transport inhibitor, or LNB167 at 32 µm or 10 µm. Nuclei were stained with SYBR Safe dye (seen in green), and cholesterol was stained with filipin reagent, in blue. Bright blue granules represent accumulated cholesterol in cells.

We performed an RNASeq experiment to search for cellular processes and pathways that would be affected by LNB167 treatment in human HEK293T cells infected or not with SARS-CoV-2 (Figure 7). RNASeq data was analyzed using the DESeq2 and Metacore softwares, to indicate significantly up- or downregulated genes and consequently, up- or down-regulated pathways in our tested conditions. A primary component analysis (PCA) of whole datasets indicated that NI and SARS-CoV-infected cells treated or not with LNB167 all clustered separately from each other (Figure 7(a)). SARS-CoV-2 infection alone resulted in upregulation of cell replication and DNA repair pathways in comparison to NI cells. Notably, several pathways related to sterol and cholesterol metabolism and biosynthesis were upregulated (Figure 7(b)). Treatment of NI cells with LNB167 resulted in the upregulation of glycosylation and anion transport programs, and also in the upregulation of membrane lipids and cholesterol metabolic and biosynthetic pathways (Figure 7(c)). In accordance, LNB167 treatment in SARS-CoV-2-infected cells indicated the upregulation of multiple pathways involving metabolism and biosynthesis of both membrane lipids and cholesterol (Figure 7(d)), which were not observed in vehicle-treated infected cells. This data indicates that LNB167 treatment causes significant changes in cellular physiology, notably in metabolic pathways involving cholesterol and membrane lipids. SARS-CoV-2 replication also involved upregulation of membrane lipid-associated pathways, suggesting that LNB167 treatment may interfere with SARS-CoV-2 dependence on host cell lipids for replication.

We used U18666A as a positive control for inhibition of intracellular cholesterol traffic as described by the supplier of the immunoassay (ABNOVA #KA1304). U18666A is a steroidal compound with reported antiviral activity against coronaviruses and other viruses [30–32] and is remarkably similar to LNB167 (Tanimoto coefficient − 0.4375) [33] (Figure S3). Since U18666A targets the intracellular cholesterol traffic, we performed an immunofluorescence assay using filipin III to visualize sterols in cellular membranes of Vero CCL81 cells infected or not with SARS-CoV-2 and treated with U18666A or LNB167 (Figure 7(e)). Our results indicated that SARS-CoV-2 infection alone provokes changes in cholesterol distribution in cells, which is in accordance with the virus ability to subvert the host cell lipid metabolism and membranes for replication [34]. U18666A treatment caused the formation of puncta inside cells that were different in comparison to dots located in the virus-infected control group and showed greater staining intensity. LNB167 treatment at 32 µM and 10 µM induced the formation of puncta that were identical to those observed in U18666A-treated cells (Figure 7(e)), regardless of whether cells were infected or not with SARS-CoV-2.

In summary, our results indicate that LNB167 affects the initial phase of SARS-CoV-2 replication, through a mechanism that likely involves modulation of intracellular cholesterol, and thereby, the properties of cellular lipid membranes.

### In vivo experiments

#### Pharmacokinetics in mice

*In vivo* pharmacokinetic studies in mice showed that LNB167 presents an oral bioavailability of 66.3%, half-life of 2.67 h, volume of distribution of 63.17 L/kg and a maximum plasma concentration of 498.69 ng/ml (1.1 µM) after a single dose of 60 mg/kg given orally (Table S2). We also evaluated the maximum tolerated dose of LNB167 by treating mice once a day for 6 days at doses of 60 or 300 mg/Kg. No signs of toxicity were observed in mice receiving the lower dose of LNB167. However, mice receiving 300 mg/Kg of LNB167 presented moderate weight loss and ruffled fur (Figure S4). In summary, we selected a dose of 120 mg/Kg once a day (q.d.) for 3 days for following *in vivo* efficacy experiments, a dose which would remain below the maximum tolerated dose observed while sustaining plasma concentrations above the EC_50_ values calculated for LNB167 *in vitro* (0.9 µM).

### Treatment with LNB167 was not protective in the hACE2-transgenic mouse model of SARS-CoV-2 infection

**Figure 8.**
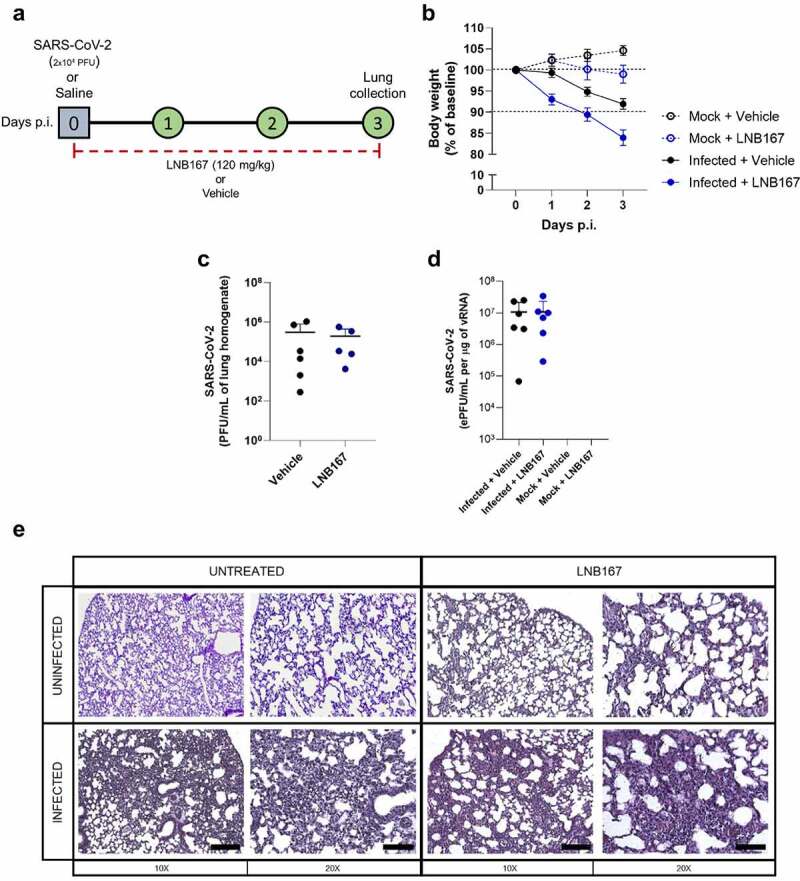
Treatment with LNB167 was not protective in the hACE2-transgenic mouse model of SARS-CoV-2 infection. K18-hACE2 TG mice were inoculated with SARS-CoV-2 (2x104 PFU/40 µl) or saline by intranasal route. Infected or uninfected animals were treated with LNB167 (120 mg/kg) or vehicle once daily from day 0 to day 3 p.I. Lungs were collected on day 3 p.I. And viral titration was performed by either (a) virus plaque forming assay or (b) RT-qPCR. (c) Body weight was evaluated daily. (d) 5-µm lung slices from mice euthanized 3 days p.I. were stained with H&E for histology assessment. Bar scales = 10x −250 µm; 20x −125 µm.

Finally, we used the transgenic mouse model of SARS-CoV-2 infection that expresses the human ACE2 receptor (hACE2) to test the protective properties of LNB167 in vivo. Adult mice were infected with SARS-CoV-2 lineage B and were treated or not with LNB167 at a dose of 120 mg/kg daily for 4 days, through gavage (Figure 8). Vehicle-treated infected mice accumulate virus in the lungs up to day 4 p.i. (Figure 8(a,b)), detectable by either plaque assay or RT-qPCR. Treatment with LNB167 did not reduce the viral load recovered from infected mouse lungs at day 4 p.i., which were similar to viral levels observed in vehicle-treated infected mice using plaque assay or RT-qPCR. Moreover, LNB167 treatment did not protect from disease development induced by SARS-CoV-2 infection in hACE2 mice (Figure 8(c,d)). SARS-CoV-2-induced weight loss in a period of 3 days p.i. was further increased by LNB167 treatment, resulting in an average weight loss of 15% in comparison to 8% observed in the vehicle-treated infected group (Figure 8(c)). Histological analysis of lung sections indicated that LNB167 treatment caused alterations in tissue architecture that were more pronounced in the context of SARS-CoV-2 infection, which are consistent with aggravation of SARS-CoV-2-induced disease (Figure 8(d)). Vehicle-treated mice, which received a solution of carboxymethylcellulose in saline by gavage, gained weight normally and showed normal lung architecture. In summary, LNB167 treatment was not effective in reducing viral load or disease development in vivo, using a transgenic mouse model of SARS-CoV-2 infection.

## Discussion

Here, we report the identification of new steroidal compounds with cationic amphiphilic properties that present antiviral activity against SARS-CoV-2 in vitro, in human cell lines. Although the COVID-19 pandemic state has been declared almost two years ago, we still do not have an effective specific treatment against the disease. Antiviral candidates originating from drug repositioning were identified (remdesivir, nitazoxanide) [35–37], along with a variety of molecules with antiviral activity, but this strategy seems exhausted [38]. New compounds are necessary for continued search for COVID-19 treatments while leading to a better understanding of viral biology and disease.

Cationic amphiphilic drugs (CADs) are characterized by their ability to disrupt phospholipid lipid homeostasis, and in the context of viral infection, may cause an antiviral effect [28,34]. The chemistry of LNB167 and LNB169, and their effects on cell biology and on SARS-CoV-2 replication indicate that these compounds are undoubtedly CADs (Figure S3). Amiodarone, chloroquine, tamoxifen, and several other CADs are drugs approved for human use that were found to exert anti-SARS-CoV-2 activity. LNB167 and LNB169 are CADs that also exert antiviral effects on SARS-CoV-2 but are new compounds and steroidal in nature. Pharmacokinetic studies performed with LNB167 (data not shown) prior to the in vivo experiments in mice (Figure 8) indicated good pharmacological properties, likely associated with the steroidal backbone. Steroidal compounds with antiviral activity against other viruses have also been described, such as tomatidine against chikungunya and dengue viruses [39]. Epiandrosterone (EA) [40,41] and 16a-bromoepiandrosterone (BrEA) [42], which are among the tested substances in this manuscript, but did not display cytoprotective activity against SARS-CoV-2 in our assays, have been tested in HIV patients [43] and led to improved antiviral immune responses, reduced expression of proinflammatory mediators and reduced viral load. Since the steroidal compounds tested in this study pertain to the androstane class, they were not expected to exert anti-inflammatory effects attributed to the pregnane class which represent the glucocorticoid family.

A mechanism of action for CADs was proposed by Kornhuber and colleagues in 2010, in which they proposed that CADs may act as functional inhibitors of acid sphingomyelinase or FIASMAs [44]. LNB167 and LNB169 are likely to be FIASMAs, as these compounds caused significant changes in cellular membrane lipid and cholesterol metabolism, as observed in RNASeq and immunofluorescence experiments (Figure 7). Function inhibition of acid sphingomyelinase (ASM) is achieved by enzyme detachment from the inner lysosomal membrane, due to accumulation of CADs in the membranes. Inhibition of ASM activity causes multiple changes in cellular physiology, including deregulation of the ceramide/sphingosine-1-P balance that regulates apoptosis/cell survival. Increased levels of sphingosine-1-p would lead to increased cell survival, which may explain why some steroidal compounds were identified as hits in cytopathic effect-based screening assays but were found to have little or no antiviral activity in validation assays (Figure 1). Moreover, increased cell survival caused by LNB167 or LNB169 FIASMA activity in non-infected cells may also explain why these compounds are not toxic in vitro in all cell types tested, and even increase relative cell viability if added to cell culture in higher concentrations (Figure S1). New experiments are required to confirm that our series of steroidal compounds are in fact FIASMAs.

Hit compound validation in Vero CCL81 cells showed that despite protecting from virus-induced CPE, compounds FFNBio41, FFNBio42, and LNB171 did not reduce viral load or viral RNA levels, in contrast to LNB167 and LNB169. We have tested our compounds in different cell-lines as a first strategy of inquiry on mechanisms of action targeting host or viral proteins, and whether compounds would retain low micromolar potency in multiple cell types. As demonstrated in Figure 3, steroidal compound action was cell-type specific, and suggested a molecular target in the host rather than the virus. Cell types used in this work represent different mammalian tissues, with expected differences in the metabolism, the composition and cellular distribution of membrane lipids and cholesterol. Such differences may explain why the anti-SARS-CoV-2 activity of LNB167 and LNB169 varied among these cell types. Furthermore, lungs, heart, kidneys, and intestines are known to express high levels of the ACE2 receptor and are susceptible to SARS-CoV-2 infection [45]. Vero cells are derived from the kidneys of African green monkeys and are widely used in virology experiments. SARS-CoV-2 spike (S) glycoprotein interacts with ACE2 in host cells and depends on the type II transmembrane serine protease (TMPRSS2) for infection [3,46]. Vero cells express the CTSL protease, which is pH-sensitive in contrast to TMPRSS2 [47]. HEK 293T is an embryonic human kidney-derived cell line commonly used to investigate mechanisms of action, which we used to perform RNASeq experiments (Figure 7)[48]. Caco-2 is a human colorectal adenocarcinoma cell line and used in ADME assays. In contrast to Vero CCL81 and HEK 293T cells, Caco-2 expresses the TMPRSS2 receptor. Calu-3 is widely used in SARS-CoV-2 *in vitro* assays as it is derived from a human lung adenocarcinoma and it has both receptors TMPRSS2 and ACE2, both important in the context of infection [3]. HuH 7.5 is a hepatic cell that also has both receptors that should be considered a suitable *in vitro* model for SARS-CoV-2 infection assays. We found no evidence to support that LNB167 or LNB169 were virucidal or inhibited viral entry, since direct incubation of the virus with these compounds had no effect on infectivity. Moreover, it is unlikely that steroidal compounds would act as direct inhibitors of the ACE2 receptor or the endosomal proteases involved in viral entry. Time of addition experiments demonstrated that compounds were most effective in the first 8 h of the replication cycle, a moment in which SARS-CoV-2 promotes the organization of double membrane vesicles and reorganizes host lipid membranes [4]. LNB167 and LNB169 affect lipid membrane and cholesterol metabolism and biosynthesis and may impair lipid membrane/cholesterol subversion by SARS-CoV-2, thus affecting viral replication.

The induction of a compound-resistant viral variant was important to understand how our steroidal compounds exert selective pressure on SARS-CoV-2 [47]. Although our findings indicate that compounds LNB167 and LNB169 modulate the host membrane lipid/cholesterol metabolism, we cannot exclude the possibility that compounds may act on viral proteins. Nonetheless, LNB167 or LNB169 treatment interfere with cell physiology, and may promote the selection of mutants and the emergence of variants with increased fitness in the outstanding conditions induced by the compounds. Our data show that complete or partial resistance could be induced and was cross protective in 169 R against LNB167. Genome sequencing corroborates showed mutations in structural proteins in resistant viral variants that are transmembrane and extensively associated with lipid membranes and cholesterol in their functions.

In conclusion, the anti-SARS-CoV-2 activities of steroidal compounds LNB167 and LNB169 are likely host-targeted, consistent with the properties of cationic amphiphilic compounds that modulate host cell lipid biology. Although effective in vitro, protective effects were cell-type specific and did not translate to protection in vivo, indicating that subversion of lipid membrane physiology is an important, yet complex mechanism involved in SARS-CoV-2 replication and pathogenesis.

## Supplementary Material

Supplemental Material

## Data Availability

The authors confirm that the data supporting the findings of this study are available within the article [and/or] its supplementary materials.
