# Supplementary material for: Identification and characterization of the anti-SARS-CoV-2 activity of cationic amphiphilic steroidal compounds
Source: Virulence. 2022 Jun 22;13(1):1031–48. doi: 10.1080/21505594.2022.2085793 (PMC9235892; doi:10.1080/21505594.2022.2085793)
Supplement: Supplemental Material [file KVIR_A_2085793_SM6119.pdf]

Supplementary results

Table S1 - Selectivity Index (SI) using half- maximun citoxicity concentration (CC_{50}) and half-minimun eficiency concetration (EC_{50}) of steroidal compounds and Chloroquine (control).

Compound	CC_{50}(µM)	EC_{50}(µM)	SI (CC_{50}/EC_{50})
**FFNBio41**	39,0	5	8
**FFNBio42**	49,3	4,7	10
**LNB149**	123,2	49,2	3
**LNB166**	53,8	21,1	3
**LNB167**	139,0	0,9	154
**LNB168**	61,3	24,6	2
**LNB169**	60,2	3,8	16
**LNB171**	68,0	18,3	4
**Chloroquine**	34,0	5,9	6

Table S2 - Mouse in vivo pharmacokinetic parameters for LNB167.

Administration route	Intravenous (6 mg/kg)	Oral (60 mg/kg)
**AUClast (h*ng/L)**	716.48	4753.87
**AUCall (h*ng/L)**	716.76	4753.87
**Tmax (h)**	0.083	0.25
**Cmax (ng/mL)**	858.64	498.69
**T1/2 (h)**	0.45	2.67
**Volume of distribution (L/kg)**	19.94	63.17
**Clearance (mL/kg/min)**	116.38	272.78
**Bioavailability (F%)**	-	66.3

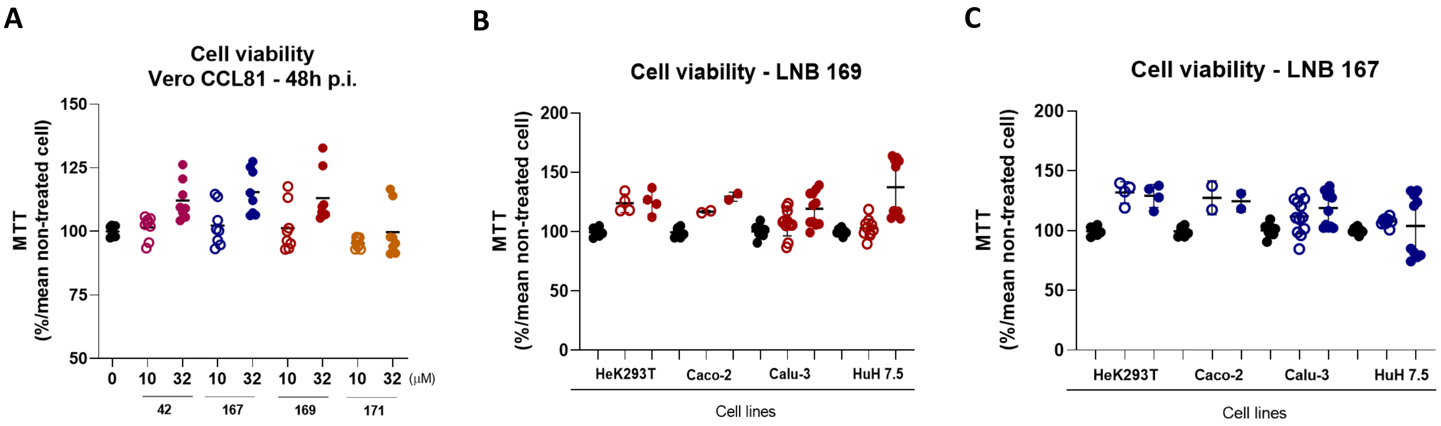

Figure S1. Steroidal compounds are not cytotoxic to cell lines used in antiviral assays. A) Steroidal compounds (FFNBio41, FFNBio42, LNB167, LNB169 and LNB171) toxicity at concentrations of 10 µM and 32 µM evaluated using the MTT assay in Vero CCL81 cells. **B)** LNB169 toxicity in different human cell lines (HEK293T, Caco-2, Calu-3 and HuH7.5) at 10 µM and 32 µM concentrations. **C)** LNB167 toxicity in human cell lines at 10 µM and 32 µM concentrations. Percentage of cell viability was calculated related with non-infected vehicle-treated cell. Vehicle: DMSO.

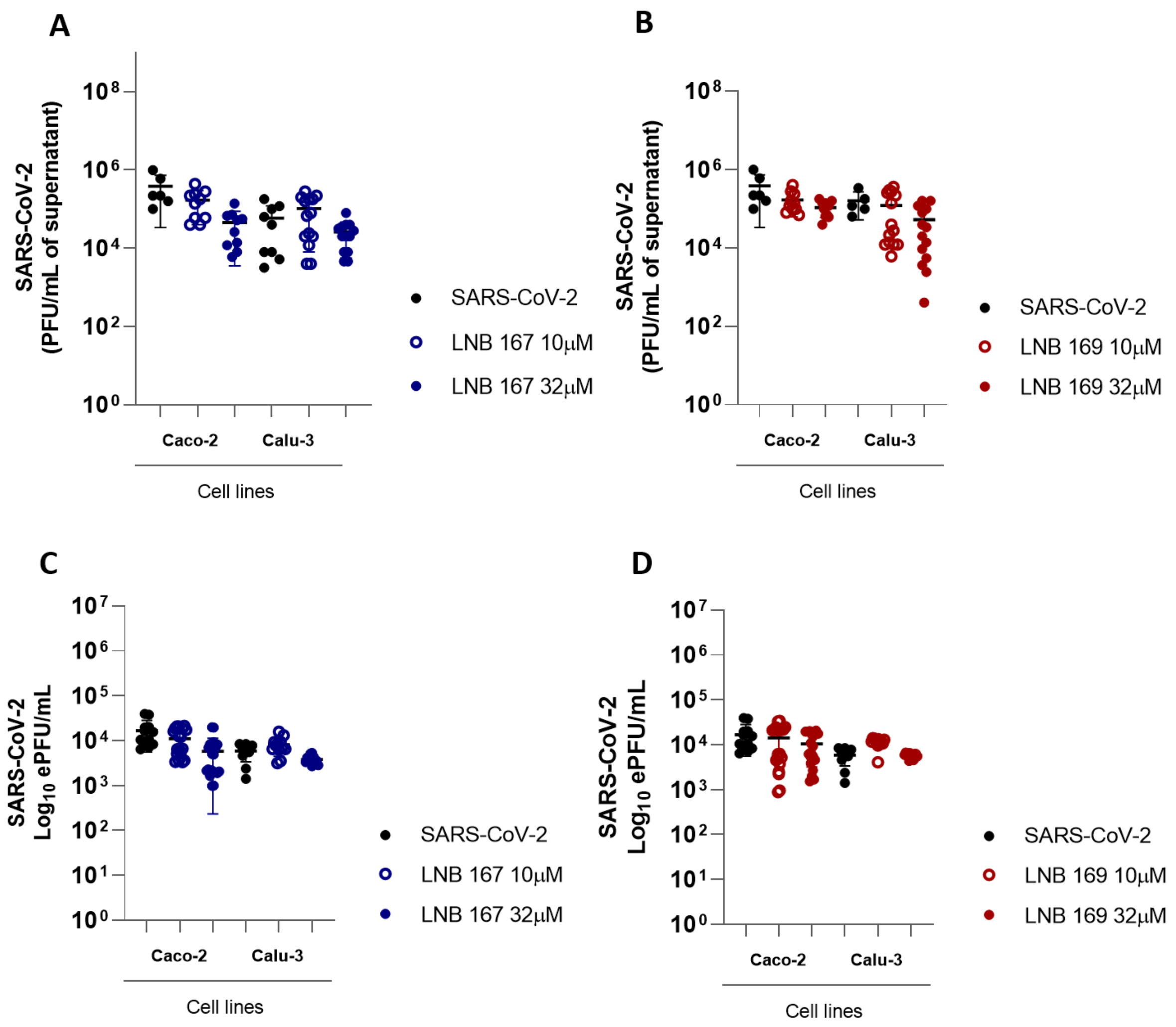

Figure S2. LNB167 and LNB169 do not have antiviral activity against SARS-CoV-2 in cell lines Caco-2 and Calu-3. (A, B) Quantification of SARS-CoV-2 infectious viral particles in the supernatant of different cell lines (Caco-2 and Calu-3) infected with SARS-CoV-2 with or without treatment, using plaque assays. **(C, D)** Viral RNA measured by RT-qPCR in supernatant of cell cultures of different cell lines infected with or without LNBio167 and LNB169 treatment. *p<0.05, **p<0.01, ***p<0.001 relative to the virus-infected, vehicle-treated control group. Vehicle = DMSO. Data representative of 2 independent experiments (n=6).

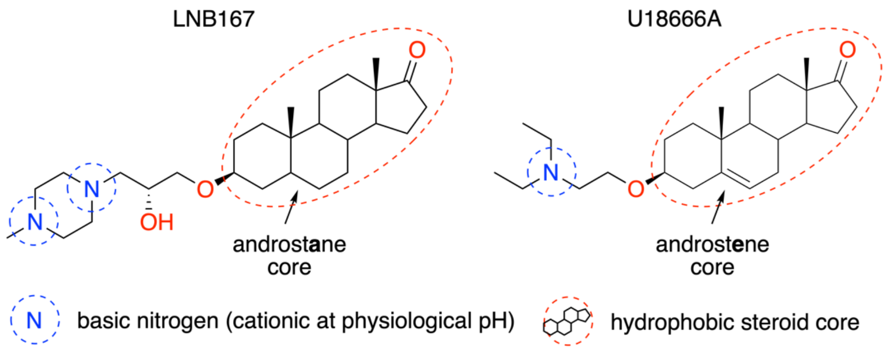

Figure S3. LNB167 and U18666A chemical structures.

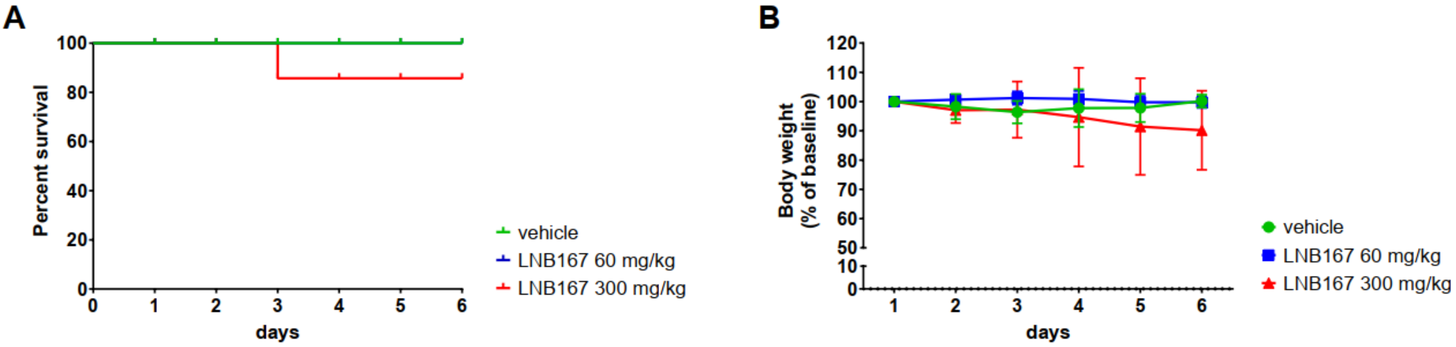

Figure S4. Evaluation of potential toxicity of LNB167 in mice. Adult FVB mice were treated daily with LNB167 at doses of 60 or 300 mg/kg or vehicle, for six days. Mice were monitored daily for signs of compound toxicity or death. (A) Mice survival during the period of six days. Death was observed in the experiment group receiving 300 mg/Kg of LNB167 due to a failed oral gavage procedure at day 3. (B) Variations in body weight normalized by mouse weigh at day 0.
